# Assessing competence of mid-level providers delivering primary health care in India: a clinical vignette-based study in Chhattisgarh state

**DOI:** 10.1186/s12960-022-00737-w

**Published:** 2022-05-12

**Authors:** Samir Garg, Narayan Tripathi, Jayathra Datla, Tomas Zapata, Dilip S. Mairembam, Kirtti K. Bebarta, C. Krishnendhu, Hilde de Graeve

**Affiliations:** 1 State Health Resource Centre, Chhattisgarh, Raipur India; 2 WHO, South East Asia Regional Office, New Delhi, India; 3 WHO, India Office, New Delhi, India

**Keywords:** Primary health care, Provider competence, Mid-level providers, Quality, Non-physician clinician, India, Non-communicable diseases, Clinical vignette

## Abstract

**Background:**

The global commitment to primary health care (PHC) has been reconfirmed in the declaration of Astana, 2018. India has also seen an upswing in national commitment to implement PHC. Health and wellness centres (HWCs) have been introduced, one at every 5000 population, with the fundamental purpose of bringing a comprehensive range of primary care services closer to where people live. The key addition in each HWC is of a mid-level healthcare provider (MLHP). Nurses were provided a 6-month training to play this role as community health officers (CHOs). But no assessments are available of the clinical competence of this newly inducted cadre for delivering primary care. The current study was aimed at providing an assessment of competence of CHOs in the Indian state of Chhattisgarh.

**Methods:**

The assessment involved a comparison of CHOs with rural medical assistants (RMAs) and medical officers (MO), the two main existing clinical cadres providing primary care in Chhattisgarh. Standardized clinical vignettes were used to measure knowledge and clinical reasoning of providers. Ten ailments were included, based on primary care needs in Chhattisgarh. Each part of clinical vignettes was standardized using expert consultations and standard treatment guidelines. Sample size was adequate to detect 15% difference between scores of different cadres and the assessment covered 132 CHOs, 129 RMAs and 50 MOs.

**Results:**

The overall mean scores of CHOs, RMAs and MOs were 50.1%, 63.1% and 68.1%, respectively. They were statistically different (*p* < 0.05). The adjusted model also confirmed the above pattern. CHOs performed well in clinical management of non-communicable diseases and malaria. CHOs also scored well in clinical knowledge for diagnosis. Around 80% of prescriptions written by CHOs for hypertension and diabetes were found correct.

**Conclusion:**

The non-physician MLHP cadre of CHOs deployed in rural facilities under the current PHC initiative in India exhibited the potential to manage ambulatory care for illnesses. Continuous training inputs, treatment protocols and medicines are needed to improve performance of MLHPs. Making comprehensive primary care services available close to people is essential to PHC and well-trained mid-level providers will be crucial for making it a reality in developing countries.

**Supplementary Information:**

The online version contains supplementary material available at 10.1186/s12960-022-00737-w.

## Background

According to the World Health Organization (WHO), primary health care (PHC) is the best approach to ensure access to healthcare with equity and efficiency [1]. The global commitment to PHC has been reconfirmed in the Declaration of Astana, 2018. Its closeness to community, ability to cover bulk of the healthcare needs of the most number of people in least cost and emphasis on equity make PHC particularly essential for low- and low- to middle-income countries (LLMICs) like India. An important component of PHC is ensuring access to curative care at primary level as it reduces mortality and need for secondary and tertiary care [2, 3].

India has also seen an upswing in national commitment to implement PHC. In 2015, a task-force was set up by the central ministry of health to provide recommendations for rolling out comprehensive PHC in India [4]. It was also a key proposal in India’s national health policy, 2017 [5]. In order to deliver PHC, an architectural correction was conceptualized in the design of public health system in India. It involved introducing health and wellness centres (HWCs), one at every 5000 population, as the hub for PHC [5]. The emergence of HWC marks a very significant development for PHC in India because of the following reasons [5–8]:

- It brings primary curative services closer to people. An HWC can be reached within half an hour by any of the families it covers.
- Earlier, the formal services available close to people were selective, largely limited to immunization and ante-natal care. As a result, for many people the unqualified informal providers were the closest option for seeking treatment when they fell sick. Now, HWCs are there to provide a wide range of services to address the primary care needs comprehensively.
- HWCs also represent a response to the epidemiological transition in India as they emphasize adequate coverage of non-communicable diseases (NCDs) at primary level.
- HWC design is based on a population health approach and to ensure a continuum of care. They aim to undertake activities for promotive and preventive health in the community, detect illnesses in time, treat the simpler conditions and refer the complicated cases to higher facilities and ensure the necessary follow-up and home-based care of chronic disease cases.

Pilots on HWCs, funded by the national health mission, were started in 2017 [9]. The central government declared HWCs as a flagship national programme in 2018 [6]. The target was to operationalize 115 000 HWCs across the country by upgrading existing facilities at 5000 population (known a sub-centres) by year 2022. By November 2021, around 51 500 of them had been made functional [10].

In order to operationalize HWCs, existing sub-centres were upgraded and services were expanded by adding infrastructure, supplies and most importantly the human resources. The key addition in staff for each HWC is that of a mid-level healthcare provider (MLHP). MLHP provides leadership to the primary care team working at the HWC and consisting of the MLHP, 1–2 paramedical staff and 5–10 community health workers [8]. MLHPs are also the main providers of healthcare to individuals coming for treatment to HWCs [8].

In order to produce and recruit MLHPs for HWCs, a policy was introduced to select nursing graduates and train them further in a bridge course of 6 months. The bridge course was designed to specifically cover the role of MLHP in HWCs [8]. This cadre trained to play the MLHP role in HWCs was designated as ‘community health officers’ (CHO).

CHOs now constitute one of the largest cadres of clinical care providers at primary level in India. But no assessments are available of the performance of CHOs including their clinical competence to detect and treat the illnesses they come across in HWCs. Provider competence can have a huge bearing on the range and quality of services the HWCs are able to provide and the amount of credibility these new institutions gain among the communities they serve. Also, if the providers are not confident in diagnosing and treating, they can end up referring most of the patients to higher facilities. That can defeat the fundamental purpose of HWCs, i.e., to bring a comprehensive range of primary care services closer to where people live [7]. The success of the HWC policy thus depends to a large extent on the competence of CHOs. The current study was therefore aimed to provide an assessment of clinical competence of CHOs. The aspect of PHC current study is focused on is of curative services at primary level.

Globally, several models have been tried to create cadres of non-physician healthcare providers suited to deliver PHC, especially for rural areas [11, 12]. These cadres have a shorter training than physicians, but are deployed to provide clinical care. The available global evidence suggests that MLHPs are performing several clinical functions that were traditionally handled by physicians [11, 13–20]. A study from India has concluded that nurses can be trained to play a clinical role in management of NCDs [21]. A systematic review has reported that when non-physicians were permitted to prescribe drugs they were able to treat patients effectively by following protocols [12]. Assessments of the performance of such non-physician healthcare providers can also be useful to inform global policies for organizing PHC [11, 12]. The current study was conducted in the state of Chhattisgarh. This state was a pioneer to start a 3-year diploma course to create a cadre of non-physician primary care providers for public facilities in rural areas in the state [22]. This cadre now has more than a decade’s experience and is known as rural medical assistants (RMA). RMA has been an important reference in design of non-physician healthcare providers in India, including the CHO cadre [4, 8]. The simultaneous presence of such cadres in Chhattisgarh offered an opportunity to conduct a comparison of CHOs with RMAs. Another useful comparison for assessing CHOs could be with medical officers (MOs) serving in primary care roles in rural areas. MOs are physicians, i.e. doctors with an undergraduate degree in medicine. A 2009 study of clinical competence of primary care providers in India had also relied on comparisons between different provider cadres [11].

## Methods and materials

### Study setting

Chhattisgarh is one of the poorest states in India. The state had a population of around 29 million and 77% of it lived in rural areas in 2020. The vulnerable indigenous communities called Scheduled Tribes constitute 31% of its population. In 2018–2019, the state had a density of 2.9 MOs per 10 000 population, which was poorer than the national average of 7.6 per 10 000 [23].

The state has 837 primary health centres, one per 30 000 population. The state had 5206 sub-centres, each covering around 5000 population and providing reproductive and child health services. By September 2020, the state had converted 1895 of its sub-centres into HWCs [24].

### Selection of providers and HWCs

The study was aimed at assessing the in-service competence of CHOs, RMAs and MOs working at the primary care level. Therefore, individuals in the above cadres working at primary health centres or HWCs for 6 months or longer were included in the study. There were 1110 CHOs, 1212 RMAs and 396 MOs fulfilling the above criteria in September 2020, when the data collection was started [23]. The minimum sample size was calculated to detect a 15% difference in mean competence scores between the groups with 90% power and confidence level of 95%. According to the above calculation, a minimum of 50 providers of each type were necessary to be covered. It was decided that around 10% of the eligible individuals in each cadre will be covered while ensuring the above minimum required sample size. The above sample size for each cadre was increased by 25% to account for non-response. Thus, a sample of 139 CHOs, 152 RMAs and 63 MOs was to be selected. The list of all 1110 CHOs working in the state was arranged district-wise from north to south. From the above list, 139 CHOs were selected using systematic random sampling. The required sample of 152 RMAs and 63 MOs was also selected through a similar procedure. The study was able to assess 132 CHOs, 129 RMAs and 50 MOs. The response rate for CHOs, RMAs and MOs was 95%, 85% and 79%, respectively.

### Study tools

Provider competence was assessed in terms of clinical knowledge for specific primary care services by using clinical vignettes. Clinical vignettes are a form of simulated clinical case structure, used primarily to measure knowledge and clinical reasoning of a healthcare provider [11, 25–27]. A key advantage of using clinical vignettes is that the case-mix is same for all the providers being assessed. This allows a valid comparison of their scores.

Similar to earlier studies, each clinical vignette was structured in the following stages—history taking, examination and investigations, diagnosis, treatment (prescription) and follow-up [11, 27]. Under each of the above stages, a set of relevant elements was added based on standard treatment guidelines and inputs from clinical experts.

The form of clinical vignettes used in this study, one of the interviewers played the part of patient and started by describing the main complaint (e.g. I am a 30-year-old woman with fever) and the provider was requested to proceed with the simulated consultation by subsequently asking questions related to history, examination and investigations. The provider was aware that the patient was imaginary and it was a simulated conversation with the interviewer. Whenever the provider asked any relevant question related to history, examination or investigation, the surveyor gave a standard response. After the history, examination and investigation sections, the provider was asked to state the diagnosis, treatment and follow-up [11, 27].

Each part of the vignette was standardized: (a) the elements expected to covered by the provider in history, examination and investigation; (b) the responses to be given by the interviewer to any relevant question by provider and c) the correct diagnosis, treatment (prescription) and follow-up care against which the providers’ responses are to be judged. The standardization was done using standard guidelines and advice of experts from the All India Institute of Medical Sciences, Raipur. The vignettes were pretested with a few CHOs, RMAs and MOs before being finalized.

### Case selection

The clinical vignettes were developed for 10 tracer conditions that cover the illnesses commonly seen at primary care level in Chhattisgarh. They were selected based on consultations with experts and practising clinicians. The clinical vignettes were on the following conditions: diarrhoea with severe dehydration, pneumonia, malaria, hypertension, diabetes, vulvo-vaginal candidiasis, pre-eclampsia, scabies, poisoning and sickle cell disease.

Some of the other important diseases in the state, like tuberculosis and leprosy, were not included as they are not expected to be diagnosed or treated at HWCs. Though HWCs do refer the presumptive cases of above diseases to higher facilities, the role does not involve clinical care.

### Scoring of clinical vignettes

The maximum score for each vignette was of 100 marks. The 100 marks for each vignette were divided across relevant elements in proportion to the relative importance of that element in ensuring the best clinical care. The element-wise distribution of marks for each vignette was decided by a set of experts. It was validated by another set of clinical experts. Other studies have also used a similar approach for deciding the marks for different parts of a clinical vignette [27–31].

### Data collection and analysis

The data collection was managed by the State Health Resource Centre, an autonomous body providing technical support to the department of health in Chhattisgarh. Each interviewer deployed for the data collection had an undergraduate degree in health sciences and a master’s degree in public health. Data collection for the study was done from October 2020 to February 2021. Apart from the vignettes, data were collected on the number of persons provided treatment by the concerned provider (CHO/RMA/MO) for various kinds of ailments. Mean and median with 95% confidence intervals were calculated for scores in different sections and vignettes. For statistical significance, one-way ANOVA was used to compare the difference in mean score of all three providers.

A multi-variate linear regression model was applied to confirm the difference in clinical scores of the three cadres. The outcome variable was the competence score achieved by the providers. We did not expect any extreme values for this variable. Five of the six independent variables included in the adjusted model were based on an existing study in Chhattisgarh [11]. The above variables were—cadre, age, sex, distance of posting place from district headquarter and type of area (tribal/non-tribal). We expected the years of experience to be a relevant variable and therefore included it in the model.

Data analysis was done using IBM-SPSS 20.0 version software.

### Ethical consideration

Ethics approval for the current study was provided by the Institutional Ethics Committee of State Health Resource Centre, Chhattisgarh, India. Informed written consent was obtained from each participant.

## Results

The socio-demographic and other characteristics of the three cadres—CHOs, RMAs and MOs doctors are given in Table 1. The proportion of women among the CHOs was 83%; as compared to 39% in RMAs and 26% in MOs. Around one-third of CHOs belonged to the vulnerable communities of Scheduled Tribes, whereas their representation among the RMAs and MOs was miniscule. The mean age of CHOs was lower than RMAs and MOs. The average distance of the workplace, i.e. the place of posting, from their native place was the closest for CHOs and farthest in case of MOs.

**Table 1:** Sample profile

Characteristic	Community health officers (CHOs)	Rural medical assistants (RMAs)	Medical officers (MOs)
	*N* = 132	*N* = 129	*N* = 50
Age category (years)			
21–25	35.6%	0.0%	12.0%
26–30	62.9%	0.8%	48.0%
31–35	0.8%	37.2%	32.0%
36–40	0.8%	58.1%	4.0%
41–45	0.0%	3.9%	4.0%
Gender			
Male	16.7%	61.2%	74.0%
Female	83.3%	38.8%	26.0%
Marital status			
Single	77.3%	5.4%	60.0%
Married	22.7%	94.6%	40.0%
Type of area where posted			
Tribal area	35.2%	39.4%	24.0%
Other area	64.8%	60.5%	76.0%
Distance of workplace from native place			
< 50 km	69.7%	61.2%	48.0%
50–100 km	8.3%	11.6%	8.0%
> 100 km	22.0%	27.1%	44.0%
Caste (social group)			
Scheduled Castes	11.4%	12.4%	16.0%
Scheduled Tribes	26.5%	3.9%	8.0%
Other Backward Classes	51.5%	51.9%	36.0%
Others	10.6%	31.0%	40.0%
Length of experience in primary care			
Mean experience in years	0.8	9.3	2.8

In terms of experience, CHOs were the least experienced. The average experience in primary care was around a year for CHOs, whereas it was close to 10 years for RMAs.

The number of persons provided treatment by the concerned provider (CHO/RMA/MO) for various kinds of ailments is given in Additional file 1: Table S1. It shows that a variety of illnesses were being treated in health and wellness centres (HWCs) of Chhattisgarh around 2019 end. Average around 359 patients received treatment per health and wellness centre in a month. HWCs managed a variety of ailments when they were staffed with a MO, RMA or a CHO.

### Competence scores

The overall competence scores of the three cadres for all the vignettes put together are given in Table 2. Overall, CHOs score less than RMAs. The MOs scored greater than both the MLHP cadres (*p* < 0.05). The median scores are given in Additional file 2: Table S2 and they show a similar pattern.

**Table 2:** Comparison of overall scores (%) between different providers (with 95% CI)

Community health officers (CHOs)	Rural medical assistants (RMAs)	Medical officers (MOs)	*P* value
*N* = 132	*N* = 129	*N* = 50	
50.1 (48.6–51.6)	63.1 (60.7–65.4)	68.1 (65.1–71.1)	< 0.01

The scores in different vignettes/diseases are given in Table 3. CHOs scored well on hypertension, diabetes and malaria and their scores for these diseases were close to what RMAs and MOs scored. CHOs scored poorly (mean score < 50%) for diarrhoea, vulvo-vaginal candidiasis and pre-eclampsia.

**Table 3:** Disease condition-wise comparison of overall scores (%) of different providers (with 95% CI)

Disease	Community health officers (CHOs)	Rural medical assistants (RMAs)	Medical officers (MOs)	*P* value
	*N* = 132	*N* = 129	*N* = 50	
Diarrhoea with dehydration	39.4 (36.5–42.5)	53.5 (50–57.1)	51.4 (45.4–57.4)	< 0.01
Chest indrawing pneumonia	50.7 (47.5–53.9)	65.3 (62.4–68.1)	64.5 (59.0–69.9)	< 0.01
Malaria	65.9 (63.4–68.5)	76.5 (74.4–78.7)	81.1 (78.1–84.1)	< 0.01
Hypertension	67.3 (64.6–70)	68.3 (65.3–71.3)	71.5 (67.3–75.7)	0.02
Diabetes	63.8 (61.2–66.4)	69.1 (66.4–71.7)	72 (68.3–75.7)	< 0.01
Vulvo-vaginal candidiasis	31.1 (27.5–34.7)	51.9 (47.8–56.1)	60.6 (53.6–67.7)	< 0.01
Pre-eclampsia	35.4 (33–37.9)	53.3 (49.8–56.7)	54.9 (49.1–60.8)	< 0.01
Scabies	48.9 (43.5–54.4)	72 (66.8–77.2)	82.4 (75.4–89.5)	< 0.01
Organo-phosphorous poisoning	48.7 (45–52.4)	61.7 (58.1–65.3)	73.5 (69.1–78)	< 0.01
Sickle cell disease	49.2 (45.5–53)	59.3 (51.9–58.7)	68.9 (63.7–74)	< 0.01

The mean scores of RMAs were above 50% for all the ten conditions but their scores for diarrhoea, vulvo-vaginal candidiasis and pre-eclampsia were poorer relative to other conditions. MOs also scored above 50% for all the ten conditions, but their scores for diarrhoea and pre-eclampsia were poorer relative to other diseases.

Table 4 gives the scores according to the component of clinical care. Among the components of clinical care, CHOs did well in diagnosis with score of 65%. Their mean scores were close to 50% in rest of the components.

**Table 4:** Clinical care component-wise comparison of scores (%) of different providers (with 95% CI)

Component	Community health officers (CHOs)	Rural medical assistants (RMAs)	Medical officers (MOs)	*P* value
	*N* = 132	*N* = 129	*N* = 50	
History taking	48 (47–50)	52 (48–55)	57 (52–61)	< 0.01
Physical examination/Investigations	43 (41–44)	48 (45–51)	56 (51–61)	< 0.01
Diagnosis	65 (62–67)	80 (78–83)	83 (79–86)	< 0.01
Treatment (prescription)	48 (46–50)	67 (64–70)	72 (68–75)	< 0.01
Follow-up	49 (47–52)	61 (58–65)	67 (60–73)	< 0.01

For RMAs and MOs, the relatively weaker areas were of history taking and physical examination/investigations. Around 80% of the prescriptions written by CHOs for hypertension and diabetes were found correct (Additional file 3: Table S3). For malaria and pneumonia, around two-thirds of prescriptions written by CHOs were found to be correct. The CHOs did not score well on prescriptions for pre-eclampsia.

The differences in overall mean scores of CHOs, RMAs and MOs on various components of clinical care were statistically significant (Table 4). MOs scored better than RMAs and CHOs. The median scores are given in Additional file 2: Table S2 and they show a similar pattern.

The adjusted multi-variate model for determinants of the overall score is given in Table 5. It showed that while controlling for the experience and other potentially relevant variables, CHOs scored less than RMAs and MOs. The rest of the variables were not associated significantly with the scores.

**Table 5:** Multi-variate linear (ordinary least squares) regression for competence score

Score	Coefficient	Std. Err	*P* value	[95% Conf. Interval]	
Age	0.010	0.035	0.785	− 0.060	0.079
Type of provider					
CHO	Reference				
RMA	1.226	0.400	0.002	0.438	2.014
MBBS	1.637	0.339	< 0.001	0.969	2.305
Gender					
Male	Reference				
Female	0.172	0.179	0.339	− 0.181	0.525
Type of area					
Tribal	Reference				
Non-tribal	0.285	0.151	0.060	− 0.012	0.582
No. of years of experience as a provider					
< 1 year	Reference				
1–3 years	0.048	0.264	0.856	− 0.471	0.567
3–6 years	0.110	0.439	0.803	− 0.755	0.974
6–9 years	− 0.251	0.353	0.477	− 0.947	0.444
> 9 years	0.014	0.287	0.962	− 0.552	0.580
Distance of HWC from district headquarter					
< 5 km	Reference				
5–15 km	− 0.083	0.447	0.853	− 0.963	0.797
16–30 km	− 0.006	0.439	0.989	− 0.871	0.859
31–45 km	0.251	0.445	0.573	− 0.625	1.127
> 45 km	0.100	0.436	0.819	− 0.759	0.959
_cons	4.414	1.070	0.000	2.306	6.521

No. of observations: 264; *R*-squared: 0.27

## Discussion

The current study provides an assessment of clinical competence of mid-level cadres in the context of PHC being expanded and organized through HWCs in India. It found that the overall competence scores of CHOs were lower than MOs and RMAs. However, the CHOs scored well in managing common NCDs and malaria. This is not surprising considering that their 6-month training programme for the MLHP role placed greater emphasis on NCDs. The programme activities in the HWCs and the system for their monitoring were also focused on NCDs. The above results indicate the potential this cadre holds in expanding access to primary care for important diseases. It underscores the need to provide them further training so as to enable them to manage a greater range of illnesses competently. It also suggests the need for modifying the monitoring design to include a wider range of diseases. The current study found several areas in which the technical skills of CHOs need to be strengthened—pre-eclampsia, reproductive tract infections, poisoning, severe dehydration and sickle cell disease. The adjusted model showed that type of cadre was significantly associated with competence scores. Equity can suffer if the difficult and underprivileged districts do not get enough MOs and have to depend mainly on mid-level cadres. This suggests that all cadres including MOs should be distributed equitably between different areas (tribal/non-tribal).

Internationally, several studies have reported that there is acceptability of non-physician prescribing; especially when the other patient-centric attributes are also present [18, 32]. Researchers have argued that prescription is a necessary part of patient-centric care and allowing non-physicians to prescribe can help in its expansion [33]. Studies have shown that prescription by non-physicians helped in timeliness of care and cost saving [19, 34, 35]. Studies have also reported that significant barriers exist in deploying MLHPs or non-physicians in clinical roles. Restrictions on prescribing are common [12]. Rigidity of boundaries, established hierarchies and relationships of power between the various medical professions has often been reported as a key barrier [34, 36]. There are often shortages of required medicines in settings where MLHPs are deployed [12].

In India, governments have made a few attempts to promote non-physician cadres though such policies have met with a significant amount of resistance from the physicians [37]. The RMA experiment remained limited to a couple of states and their numbers remained below 2000 [11, 37, 38]. The Bachelor of Rural Health Care (BRHC) course was proposed by the central ministry of health in 2010, but it could not be launched due to sustained opposition [39]. The course was redesigned as Bachelor of Science (Community Health) and it got the central government’s approval in 2013 [39, 40]. Yet, it remained largely unimplemented and only one or two states could make a start [41]. In comparison to the earlier attempts, CHO cadre has gone the most distance. Many countries seem to be shifting to nurse-based MLHPs. A similar shift could also be seen in the Indian policy, from non-nurse MLHPs like RMAs to nurse-based cadre of CHOs. There are more than 50 000 CHOs already working and their number is likely to cross 100 000 soon. CHOs are perhaps facing less opposition as they are posted in small rural facilities (sub-centres). It is important to note that the policy does not allow CHOs to practise outside HWCs. While earlier attempts to promote MLHPs were stand alone in nature, CHOs were part of the comprehensively designed mechanism of HWCs. This suggests that system-wide amendments, like introduction of HWCs may be necessary for such cadres to get established. Another factor that seems to have facilitated the fast roll-out of CHO cadre is of increased production of nurses in India and availability of surplus nurses.

HWCs are emerging as a suitable vehicle to implement PHC in India as they aim to bring comprehensive services closer to rural people, at a population of 5000. There is no way physicians can be placed at such a grassroots level in the foreseeable future [42]. Still there can be a tendency to restrict the role of HWCs in conducting diagnosis and treatment of illnesses and to confine their involvement to screening, referral and follow-up. Such a tendency is likely to have an adverse affect on PHC. The patients referred by HWCs to higher facilities may have to travel large distances and it can involve a lot of difficulty and uncertainty in most parts of India. Therefore, if HWCs have to fulfil their relevance in PHC, MLHPs need to treat a large share of the patients approaching them and limit the referrals mainly to complicated illnesses. A recent policy brief in Indian context has also recommended expanding the prescription rights of nurses [43]. The development of CHO cadre and their acceptability in curative ambulatory care role can perhaps help in improving the status of nurses in Indian health system. CHOs can also help in correcting some of the gender imbalance in clinical cadres in India.

While a long-standing debate exists on how far to rely on non-physician cadres for clinical roles, the advantages they offer should not be ignored any longer, especially in LLMIC situations like India’s. The results of the current study bode well for the CHO cadre deployed in HWCs. The question that requires attention now is how to enable such cadres to ensure the optimal delivery of PHC. This also underscores the need for ensuring adequate in-service training and continuous skill building for such cadres. Earlier studies have shown that continuous training is necessary and effective in improving clinical performance of non-physician cadres in PHC [31, 44]. It is significant therefore that the national health mission in India has recently initiated programmes for in-service capacity building of CHOs [45].

Development of standard treatment protocols has also been recommended to enable the non-physician cadres in providing better quality care [12]. A specific recommendation has been to develop simplified protocols for NCD management in facilities including the HWCs [46]. Chhattisgarh has also developed standard treatment protocols for use by CHOs and they form an important aid for building greater clinical competence [47].

The current study has several strengths. It was the first study on clinical competence of CHOs and it provides important guidance for policies to expand PHC in rural areas. The study covered a wide range of ambulatory care competencies and was not limited to a single disease. The study identifies areas in which the technical capacities of CHOs need to be enhanced. There will be a need to conduct similar studies of CHOs in other Indian states. A qualitative study is recommended to understand how the CHO cadre got developed. Further research is recommended on health outcomes achieved by MLHPs in HWCs, the cost-effectiveness of care provided by them and impact of their introduction on the wider health system.

### Limitations

The limitations of clinical vignettes-based assessments apply: (a) performance on the vignettes can be different from what providers do in practice; (b) this does not include demonstrating the skills to perform the clinical tasks necessary to diagnose and care for a patient. It is difficult to directly compare the scores of MLHPs in this study with other studies because the vignettes and their scoring templates vary across studies.

## Conclusion

The non-physician MLHP cadre of CHOs deployed in rural facilities under the current PHC initiative in India exhibited the potential to manage ambulatory care for illnesses. They should be trained further so that they can treat a large share of patients coming to the health and wellness centres. Their training should also equip them for appropriate referrals to higher facilities based on assessment of likely complications.

Continuous training inputs, treatment protocols and medicines need to be made available to MLHPs to improve their performance. Making a wide range of primary care services available close to people is essential to PHC and well-trained mid-level providers will be crucial for making it a reality.

## Supplementary Information

**Additional file 1: Table S1.** No. of persons (mean) treated in health and wellness centres per month for various ailments—by type of provider.

**Additional file 2: Table S2.** Overall median scores of different providers in % (with 95% CI).

**Additional file 3: Table S3.** Disease wise scores in prescription (treatment) for providers (% with 95% CI).

## Data Availability

The datasets used and/or analysed during the current study are available from the corresponding author and State Health Resource Centre, Chhattisgarh on reasonable request.

## Abbreviations

CHO: Community health officer
HRH: Human resources for health
HWC: Health and Wellness Centre
LLMIC: Low- and low- to middle income country
MBBS: Bachelor of Medicine and Bachelor of Surgery
MLHP: Mid-level healthcare provider
MO: Medical officer
NCD: Non-communicable disease
PHC: Primary Health Care
RMA: Rural medical assistant
UG: Under graduate
UHC: Universal Health Coverage
WHO: World Health Organization
