# Supplementary material for: Assessing competence of mid-level providers delivering primary health care in India: a clinical vignette-based study in Chhattisgarh state
Source: Hum Resour Health. 2022 May 12;20:41. doi: 10.1186/s12960-022-00737-w (PMC9097044; doi:10.1186/s12960-022-00737-w)
Supplement: Supplementary file 1 — Additional file 1: Table S1. No. of persons (mean) treated in health and wellness centres per month for various ailments—by type of provider. [file 12960_2022_737_MOESM1_ESM.docx]

**Additional file 1**

**Table S1: No. of persons (mean) treated in health and wellness centres per month for various ailments– by type of provider**

| **Disease/Condition** | **No. of persons treated per Health and Wellness Centre in one month** | | |
| --- | --- | --- | --- |
|  | **CHO** | **RMA** | **MO** |
| **Reproductive and Child Health** |  |  |  |
| Ante-natal care | 24 | 32 | 30 |
| Deliveries | 3 | 6 | 8 |
| Newborn illness | 1 | 3 | 3 |
| **Communicable Diseases** |  |  |  |
| Diarrhea | 16 | 13 | 18 |
| ARI/Pneumonia | 1 | 3 | 4 |
| Tuberculosis follow up | 2 | 3 | 4 |
| Reproductive tract infection | 3 | 5 | 5 |
| Skin infection | 24 | 42 | 48 |
| Ear infection | 5 | 9 | 10 |
| Eye infection | 3 | 9 | 10 |
| Malaria | 4 | 5 | 5 |
| **Non-Communicable diseases** |  |  |  |
| Hypertension | 40 | 30 | 39 |
| Diabetes | 27 | 23 | 31 |
| Sickle cell disease | 0 | 0 | 2 |
| Epilepsy | 0 | 0 | 1 |
| **Emergency care/Injuries** |  |  |  |
| Minor injuries/burns | 13 | 21 | 24 |
| Severe dehydration | 1 | 2 | 3 |
| **Other acute ailments** |  |  |  |
| Fever | 50 | 56 | 64 |
| Cold and cough | 46 | 44 | 53 |
| Aches and pains | 47 | 43 | 53 |
