# Supplementary material for: Assessing competence of mid-level providers delivering primary health care in India: a clinical vignette-based study in Chhattisgarh state
Source: Hum Resour Health. 2022 May 12;20:41. doi: 10.1186/s12960-022-00737-w (PMC9097044; doi:10.1186/s12960-022-00737-w)
Supplement: Supplementary file 2 — Additional file 2: Table S2. Overall median scores of different providers in % (with 95% CI). [file 12960_2022_737_MOESM2_ESM.docx]

**Additional file 2 – Median Scores of different providers**

**Table S2: Overall Median Scores of different providers in % (with 95% CI)**

|  | | **CHO** | | | | **RMA** | | **MO** | | |
| --- | --- | --- | --- | --- | --- | --- | --- | --- | --- | --- |
|  | | N=132 | | | | N=129 | | N=50 | | |
| **All** | | 51.0  (49.6-53.2) | | | | 63  (62.3-66.4) | | 67.4  (64.3-74.6) | | |
| **Disease wise Median Scores (%) of different providers (with 95% CI)** | | | | | | | | | | |
| Diarrhea with dehydration | | | 37  (34.6-39.6) | | 53.4  (48.1-60.2) | | | | 51.1  (44.0-61.1) | |
| Chest Indrawing Pneumonia | | | 50.2  (46.6-54.3) | | 66.6  (62.8-69.2) | | | | 65.4  (59.6-70.1) | |
| Malaria | | | 68.1  (64.7-71.3) | | 79.1  (76.0-81.7) | | | | 82.9  (76.9-85.6) | |
| Hypertension | | | 69.7  (65.9-72.5) | | 69.7  (67.4-50) | | | | 74.1  (66.2-80.4) | |
| Diabetes | | | 65.8  (64.0-68.7) | | 72.4  (67.0-74.7) | | | | 72.8  (67.3-79.9) | |
| Vulvovaginal Candidiasis | | | 26.0  (23.5-30.6) | | 55.2  (44.3-60.5) | | | | 64.0  (54.8-71.7) | |
| Pre-eclampsia | | | 34.8  (32.1-37.3) | | 53.6  (50.9-58.1) | | | | 53.9  (44.1-63.7) | |
| Scabies | | | 54.1  (41.7-66.6) | | 83.3  (83.3-84.3) | | | | 91.6  (87.7-91.6) | |
| Organo-phosphorous Poisoning | | | 48.1  (45.0-52.4) | | 62.1  (57.9-69.6) | | | | 75.8  (66.0-81.4) | |
| Sickle Cell Disease | | | 51.0  (49-53.1) | | 61.1  (57.7-63.2) | | | | 69.1  (65.1-74.0) | |
| **Component of care wise Median Scores (%) of different providers** | | | | | | | | | | |
| History | | 48  (46-52) | | | 50  (46-56) | | | 58  (48-67) | | |
| Physical examination/ Investigation | | 43  (41-44) | | | 48  (43-53) | | | 58  (52-67) | | |
| Diagnosis | | 65  (65-70) | | | 85  (80-85) | | | 87  (83-90) | | |
| Treatment | | 48  (46-50) | | | 68  (64-71) | | | 74  (66-77) | | |
| Follow up | | 50  (47-52) | | | 61  (57-67) | | | 66  (56-76) | | |
