# Supplementary material for: Assessing competence of mid-level providers delivering primary health care in India: a clinical vignette-based study in Chhattisgarh state
Source: Hum Resour Health. 2022 May 12;20:41. doi: 10.1186/s12960-022-00737-w (PMC9097044; doi:10.1186/s12960-022-00737-w)
Supplement: Supplementary file 3 — Additional file 3: Table S3. Disease wise scores in prescription (treatment) for providers (% with 95% CI). [file 12960_2022_737_MOESM3_ESM.docx]

|  | **CHO** | **RMA** | **MO** | **P value** |
| --- | --- | --- | --- | --- |
| **Disease** | N=132 | N=129 | N=50 |  |
| Diarrhea with dehydration | 31.6  (26.5-36.7) | 43.9  (38.1-49.7) | 41.7  (32.5-50.9) | <0.01 |
| Chest In-drawing Pneumonia | 64.4  (59.7-69.1) | 79.20  (74.9-83.5) | 73.3  (64.85-81.8) | <0.01 |
| Malaria | 65.0  (60.1-69.9) | 86.2  (82.3-90.1) | 91.6  (86.4- 96.8) | <0.01 |
| Hypertension | 83.4  (80.2-86.6) | 81.92  (77.9-85.9) | 87.1  (81.5-92.7) | 0.330 |
| Diabetes | 79.0  (75.0-83.0) | 85.0  (81.4-88.5) | 89.1  (84.1-94.1) | 0.008 |
| Vulvo-vaginal Candidiasis | 35.34  (28.7-42.0) | 62.0  (55.8-68.1) | 74.6  (65.0-84.3) | <0.01 |
| Pre-eclampsia | 14.1  (8.8-19.5) | 63.5  (55.9-71.2) | 46.7  (33.9-59.4) | <0.01 |
| Scabies | 41.9  (36.9-46.9) | 64.2  (59.5-68.8) | 77.0  (70.1-83.8) | <0.01 |
| Organo-phosphorous Poisoning | 41.4  (36.9-46.0) | 53.5  (48.5-58.5) | 67.7  (61.2-74.2) | <0.01 |
| Sickle Cell Disease | 35.0  (31.7-38.3) | 51.2  (46.7-55.6) | 66.4  (59.5-73.3) | <0.01 |

**Additional file 3**

**Table S3: Disease wise scores in Prescription (treatment) for providers (% with 95% CI)**
